# Transcriptional Analysis of Sepsis-Induced Activation and Damage of the Adrenal Endothelial Microvascular Cells

**DOI:** 10.3389/fendo.2019.00944

**Published:** 2020-01-22

**Authors:** Lan-Sun Chen, Sumeet P. Singh, Gregor Müller, Stefan R. Bornstein, Waldemar Kanczkowski

**Affiliations:** ^1^Institute of Medical Microbiology and Hygiene, Technische Universität Dresden, Dresden, Germany; ^2^Department of Internal Medicine III, University Hospital Carl Gustav Carus at the Technische Universität Dresden, Dresden, Germany; ^3^IRIBHM ULB, Brussels, Belgium

**Keywords:** the HPA axis, RNAseq, adrenal dysfunction, microvascular endothelial cells, sepsis

## Abstract

Bacterial sepsis is a serious threat to the body homeostasis and is often associated with high mortality in non-coronary intensive stations. In order to survive sepsis, rapid activation of the hypothalamus-pituitary-adrenal gland axis and sympathomedullary system is necessary. In many patients with sepsis, the function of those two arms of the stress system is dysregulated with underlying mechanisms remaining unknown. In our previous experimental studies, we have demonstrated that LPS-induced systemic inflammation and CLP-induced peritonitis can result in adrenal gland damage. Histological and transcriptomic analysis revealed a potential involvement of the adrenal microvascular endothelium in this process. However, our knowledge about the function of adrenal microvascular cells during sepsis is scarce. In the present study, we have characterized transcriptomic alterations in isolated mouse adrenal microvascular endothelial cells induced by systemic administration of bacterial LPS. Our results revealed that LPS induced a distinct transcriptomic profile in the adrenal microvascular cells, including multiple genes regulating inflammation, activation of the coagulation cascade and vascular permeability. Activation of those genes may be potentially involved in the damage to the microvascular endothelium and altogether contribute to the sepsis-mediated adrenal dysregulation.

## Introduction

Sepsis and septic shock are major causes of death in non-coronary intensive care stations worldwide. Despite decades of intensive basic and clinical research, currently, no specific therapeutic interventions are available and the treatment of patients with sepsis is mostly focused on support of their organ function (1). Sepsis is characterized by progressive dysfunction of multiple organs as a result of the improper host response to systemic microbial infection (2). The mechanisms involved in the latter process are complex, multifactorial, and mostly unexplored (3).

It is generally accepted that prompt activation of the hypothalamic-pituitary-adrenal (HPA) axis and the sympathomedullary system (SAS), known collectively as the stress system, is crucial in surviving sepsis (4). Many patients with a prolonged stay at the intensive care stations develop adrenal gland dysfunction, which results from an early inhibition of pituitary ACTH secretion due to dysfunctional glucocorticoid hormone metabolism and prolonged activation of the immune-adrenal crosstalk (5, 6). The latter factors may increase the risk of sepsis-related death among those patients (7, 8). However, the diagnosis of the adrenal gland insufficiency and identification of patients that may benefit from glucocorticoid therapy remain as enigmatic as sepsis syndrome itself (9).

In the last years, our group has been investigating potential mechanisms involved in the sepsis mediated adrenal gland dysregulation (4). We have demonstrated that during both LPS- and cecal-ligation and puncture (CLP)-induced sepsis, adrenal gland damage occurs, including a rapid increase in intraadrenal inflammation, enhanced cell death of adrenal cells and occurrence of hemorrhages (10, 11). In our latest study, we have characterized the adrenal gland transcriptome changes induced by LPS using the RNA sequencing technique. Results of that investigation demonstrated that LPS induced a strong inflammation in the adrenal gland along with hypoxia and the coagulation pathway, which suggests the potential involvement of the adrenal microvasculature (12).

In non-stressed conditions, tissue microvascular endothelial cells are involved in the maintenance of tissue homeostasis. In particular, these cells provide an essential barrier between circulation and parenchymal cells, control vascular tone, the coagulation properties of blood, and regulate leukocyte recruitment (13). However, during sepsis, this homeostatic function of the endothelial cells is often dysfunctional (14). In those conditions, elevated plasma and local concentration of proinflammatory cytokines activate endothelial cells, resulting in an increased expression of P- and E-selectins, chemokines, and adhesion molecules e.g., ICAM-1 or VCAM-1 by these cells. As a result, increased infiltration of immune cells triggers inflammation in affected organs (15). Moreover, during activation, endothelial cells enter often a procoagulant phase, which is associated with increased expression of tissue factor and plasminogen activator inhibitor-1 (PAI-1) and decrease in anticoagulants level, including activated protein C or thrombomodulin. This situation together with increased permeability of vasculature and disruption in endothelial barrier integrity often predisposes to hemorrhages (16).

Although vascular damage is undisputedly involved in the sepsis-induced dysfunction of many organs, scarce information is available regarding the adrenal gland vascular endothelial cells (17, 18). Therefore, the main purpose of this study was to study the potential characteristics of microvascular damage by performing a next-generation sequencing analysis of transcriptome changes in adrenal microvascular endothelial cells isolated from mice with LPS-induced systemic inflammation.

Our results, clearly demonstrate that the adrenal microvascular endothelial cells may actively contribute to sepsis-induced adrenal dysfunction. In particular, we have found that sepsis promotes the expression of several genes involved in vascular cell inflammation, leakage, and coagulation, which may ultimately contribute to adrenal gland hemorrhages, and hypoxia.

## Materials and Methods

### Animals

C57BL/6JRj male mice were purchased from the Javier Labs (France). Mice were divided into two groups. A systemic inflammatory response syndrome (SIRS) group (*n* = 11) and a control group (*n* = 11). Both groups were injected intraperitoneally at the age of 10 weeks, either with 1 mg/kg body weight of bacterial LPS (serotype 0111: B4; Invivogen; France) in the SIRS group or with 0.9% NaCl (physiological saline)—in the control group. Three hours after LPS injection mice were killed and adrenals were excised for further analysis. The experiment was approved by the German ethical committee of the Landesdirection Dresden.

### Isolation of Mouse Adrenal Gland Endothelial Cells

For isolation of endothelial cells, adrenal glands were digested using a solution containing collagenase I and bovine serum albumin (both at 1.6 mg/ml concentration, Sigma-Aldrich, Germany) dissolved in the phosphate-buffered saline (PBS). Digestion was performed for a total of 30 min at 37°C in a thermomixer with shaking. After digestion, cells were dissociated using a 1 ml tuberculin syringe and 20 Gauge needle (Braun, Germany) and by subsequent passing through 100 μm-pore size strainers. Resulting cell suspensions were centrifuged at 2,900 RPM for 8 min at 4°C, pelleted, and washed in a FACS buffer (PBS solution containing 5% of fetal calf serum). Afterward, cells were incubated for 60 min at 8°C with a mixture of conjugated antibodies in the FACS buffer: including rat against mouse CD45-PE antibody (immune cells), rat against mouse CD31-PE/CY7 antibody (endothelial cells) and rat against mouse Ter119-APC (erythrocytes) antibody. All those monoclonal antibodies were purchased from BD Bioscience (BD Biosciences, USA). Dead cells were excluded using Hoechst 33258 (Invitrogen, Thermo Fisher Scientific). Populations of single, alive, CD31 positive endothelial cells were subsequently isolated by BD FACSAria III sorter (BD Biosciences). From each adrenal gland, 30.000 endothelial cells were sorted out and used for further analysis.

### RNA Isolation and Quantitative PCR Analysis

RNA isolation was performed using the RNeasy Plus Micro Kit (Qiagen, Germany), according to a manufacturing protocol. High quality and integrity of RNA samples (*n* = 3 per group) used for the RNA-Sequencing experiment were additionally verified by a Bioanalyzer 2100 (Agilent, USA). Samples used for PCR validation (*n* = 5 per group), were reverse-transcribed using the iScript cDNA Synthesis Assay (Bio-Rad, Germany) in a final volume of 20 ml according to manufacture protocol. Real-time PCR, which was performed using SsoFast Eva Green Supermix (BioRad) and previously reported gene-specific primers (12) in a CFX96 Real-Time PCR detection system (BioRad). Gene expression was calculated based on the ΔΔCt method upon normalization with 18S rRNA gene (19).

### Libraries Preparation for RNA Sequencing and Extraction of Data

RNA sequencing was performed by the Deep Sequencing Facility Group (BIOTEC, Center of Regenerative Therapies Dresden, Germany). The procedure of data extraction and analysis has been described in detail in our previous study (12). Briefly, fastq formatted raw reads were trimmed using a “trim-galore” package with default settings for adapter sequences removal (12). Trimmed data were further mapped to the mouse genome GRCm38 by using HISAT2 (20) with default parameters. Htseq-count (21) was used to assign reads to exons thus eventually getting counts per gene. Quality control, normalization, and scaling of the raw read counts were performed by EdgeR (22) package as a part of an integrated Differential Expression and Pathway analysis (iDEP.90) web-based tool for analyzing RNA-seq data (23). Differentially expressed genes were identified using the following criteria: minimum 50 counts per million (CPM) in at least three libraries, an adjusted *p*-value (padj) < 0.05 and minimal fold change > 2. Unsupervised analysis of the 100 most variable genes between treatments was performed using a cluster analysis based on Euclidean distance as a part of the iDEP.90 software.

### Gene Ontology Analysis

A number of top 500 upregulated (UP) and top 500 downregulated (DOWN) genes were submitted to the DAVID software (version 6.7, https://david-d.ncifcrf.gov) for analysis of gene ontology and functional mapping (24). An EASE cut off score of *p*-value < 0.05 was chosen for gene enrichment in annotation terms (25). *P*-values from enriched pathways or GO terms were transformed into—log 10 and visualized by pyramid slot transformations.

### Gene Set Enrichment Analysis (GSEA)

All significantly changed genes were subjected to GSEA software v4.0.1 from the Broad Institute (http://www.gsea-msigdb.org/gsea/index.jsp) (26) for pathway enrichment and functional annotation analysis. GSEA was performed with a default setting, and annotated Hallmarks gene sets collection v7.0, from Molecular Signatures Database (MSigDB, http://software.broadinstitute.org/gsea/msigdb/index.jsp), was used as an enrichment database. Gene sets with nominal *p*-value < 0.05 and FDR *q*-value < 0.25 were considered as enriched and were further investigated.

### Immunofluorescent Staining

Adrenal glands were cleaned from surrounding fat and fixed in a 4% paraformaldehyde solution for 1 h. After washing, adrenals were incubated in increasing concentrations (5–30%) of sucrose solution, embedded in Tissue-Tek® O.C.T.™ Compound (Sakura, Japan) and kept at −80°C. For immunofluorescent staining, 6 μm-thick tissue slices were incubated for 1 h in a blocking solution (composed of 0.3% Triton 100 and 5% normal goat serum in PBS) and then incubated overnight at +8°C in a buffer containing antigen-specific primary antibodies (composed of 0.3% Triton 100 and 5% BSA in PBS). The following antibodies were used: polyclonal rabbit antibody anti-mouse thrombomodulin (Thbd, 1:800, ab130152, Abcam), monoclonal rat anti-mouse CD31 antibody (1:100, ab7388, Abcam), monoclonal rat anti-mouse CD34 antibody conjugated with 488 dye (1:50, 11-0341-82, eBioscience) and monoclonal rabbit anti-mouse VCAM-1 antibody (1:200, clone EPR5047, ab134047, Abcam, USA). Primary antibodies were then detected by secondary antibodies conjugated to either Cy3 or 488 fluorescent dyes (VCAM-1, CD31, Thmd1) and counterstained with a nuclear dye (DAPI, 1:10000). Isotype controls were used as controls for CD146 and CD34 antibodies. Pictures were acquired with a × 10 objective using the Axio Imager 2 light microscope (Carl Zeiss, Jena, Germany).

### Protein Extraction and Western Blot

For protein isolation, adrenal glands were briefly sonicated in a 1x lysis buffer containing a proteinase/phosphatase inhibitor cocktail (both buffers were from Cell Signaling Technology, USA). Twenty micrograms of proteins were separated on 8% polyacrylamide gel and subsequently blotted onto polyvinylidene fluoride (PVDF) membrane. Membranes were then incubated for 120 min in a blocking solution composed of non-fat milk (5%) dissolved in TBS-T (tris buffered saline buffer containing Tween®20 detergent). For the VCAM-1 and GAPDH detection following antibodies were used rabbit monoclonal anti-mouse VCAM-1 antibody (1:1000, clone EPR5047, ab134047, Abcam), a monoclonal rabbit anti-mouse GAPDH (1:1000; clone 14C10, #2118, Cell Signaling Technology), and goat anti-rabbit HRP conjugated secondary antibodies (1:6000, CST). The expression of VCAM-1 protein was analyzed based on densitometry in ImageJ software (http://rsb.info.nih.gov/ij/index.html).

### Protein Cytokine Array

Six adrenals from each experimental group were isolated and immediately homogenized in 500 μl PBS containing protease inhibitor cocktail (P8340, Sigma-Aldrich, Merck). After homogenization, Triton X-100 was supplemented to a total concentration of 1%, and samples were frozen at −80°C. After thawing on ice and centrifuging at 13,000 rpm for 5 min supernatants were collected. Protein concentration was determined and 300 μg of each lysate was applied to the Proteome Profiler Mouse Cytokine Array Kit, Panel A (R&D Systems, catalog ARY006). The chemiluminescence reaction was measured with Syngene G: BOX XT4: Chemiluminescence and Fluorescence Imaging System (Integrated Scientific Solutions, Inc. USA). Densitometry quantitation was performed using FIJI/ImageJ software (NIH, USA).

## Results

### Identification of the Adrenal Gland Microvascular Endothelial Cells

**Figure 1:**
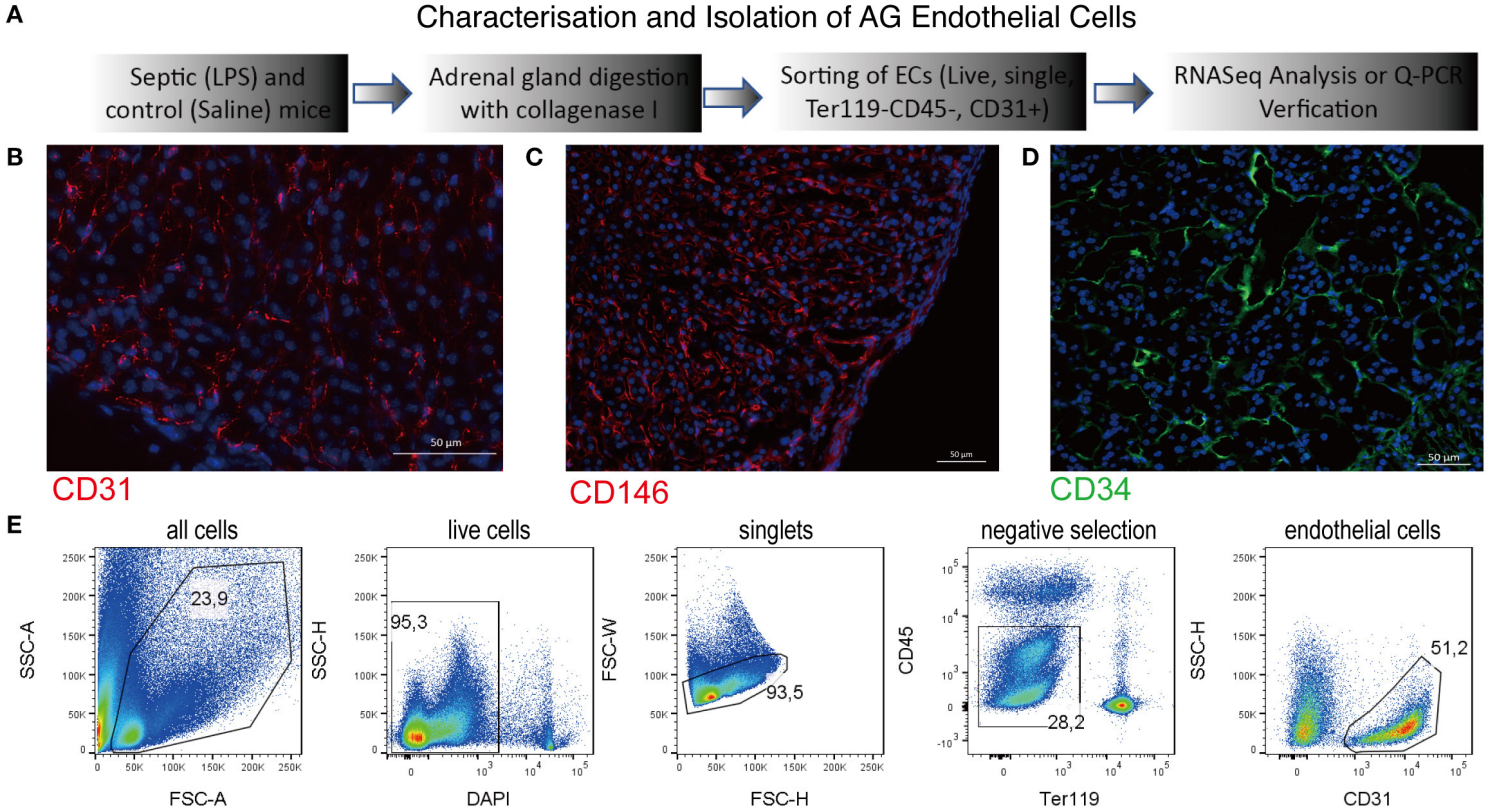
Characterization of the adrenal gland microvascular endothelial cells. Schematic representation of the procedures used to isolate and to characterize the adrenal microvascular endothelial cells **(A)**. Fluorescent staining of the mouse adrenal gland sections for endothelial-specific markers such as CD31 (PECAM) **(B)**, CD146 (MCAM) **(C)**, and CD34 **(D)**. CD31 and CD146 were visualized using secondary antibodies coupled to Cy3 dye (red color), and CD34 was visualized using secondary antibodies coupled to 488 dye (green color). Representation of the multiparametric FACS sorting technique used to isolate adrenal microvascular endothelial cells **(E)**. The gating strategy was based on negative selection. Only alive, single cells that were CD45 negative (devoid of immune cells) and Ter119 negative (devoid of erythrocytes) but CD31 positive (endothelial cells) were sorted.

In order to study transcriptional changes of the adrenal vascular cells, we had to first identify an efficient method that can provide us an exact and high number of alive, single endothelial cells that can be subsequently used for high-quality RNA isolation. Since techniques based on antibody-coated magnetic beads often provide mixed populations of endothelial cells containing also parenchymal and perivascular cells (27), we have decided to use multiparametric flow cytometry sorting. In a search of a specific and stably expressed cell membrane marker, we have performed a series of immunofluorescent staining in adrenal gland tissue sections and a FACS validation of primary cells. We have verified a specific expression of the following endothelial markers in mouse adrenal glands: a cluster of differentiation (CD) member 31, known also as platelet endothelial cell adhesion molecule (PECAM) (Figure 1B), melanoma cell adhesion molecule (MCAM; CD146) (Figure 1C) and CD34 (Figure 1D). Afterwards, we have tested the expression of those markers by FACS in adrenal cells from control and SIRS groups. Based on this analysis, we have chosen the CD31 marker for isolation of endothelial cells from the adrenal gland due to its high and stabile expression among control and SIRS groups. Therefore, in our further studies, we will refer to adrenal microvascular endothelial cells as primary cells expressing CD31, which are negative for pan immune cell marker, CD45, and are devoid of erythrocytes (Ter119-negative; Figure 1E).

### Bioinformatics Analysis of the Adrenal Microvascular Endothelial Cell Transcriptome During LPS-Induced Systemic Inflammation

**Figure 2:**
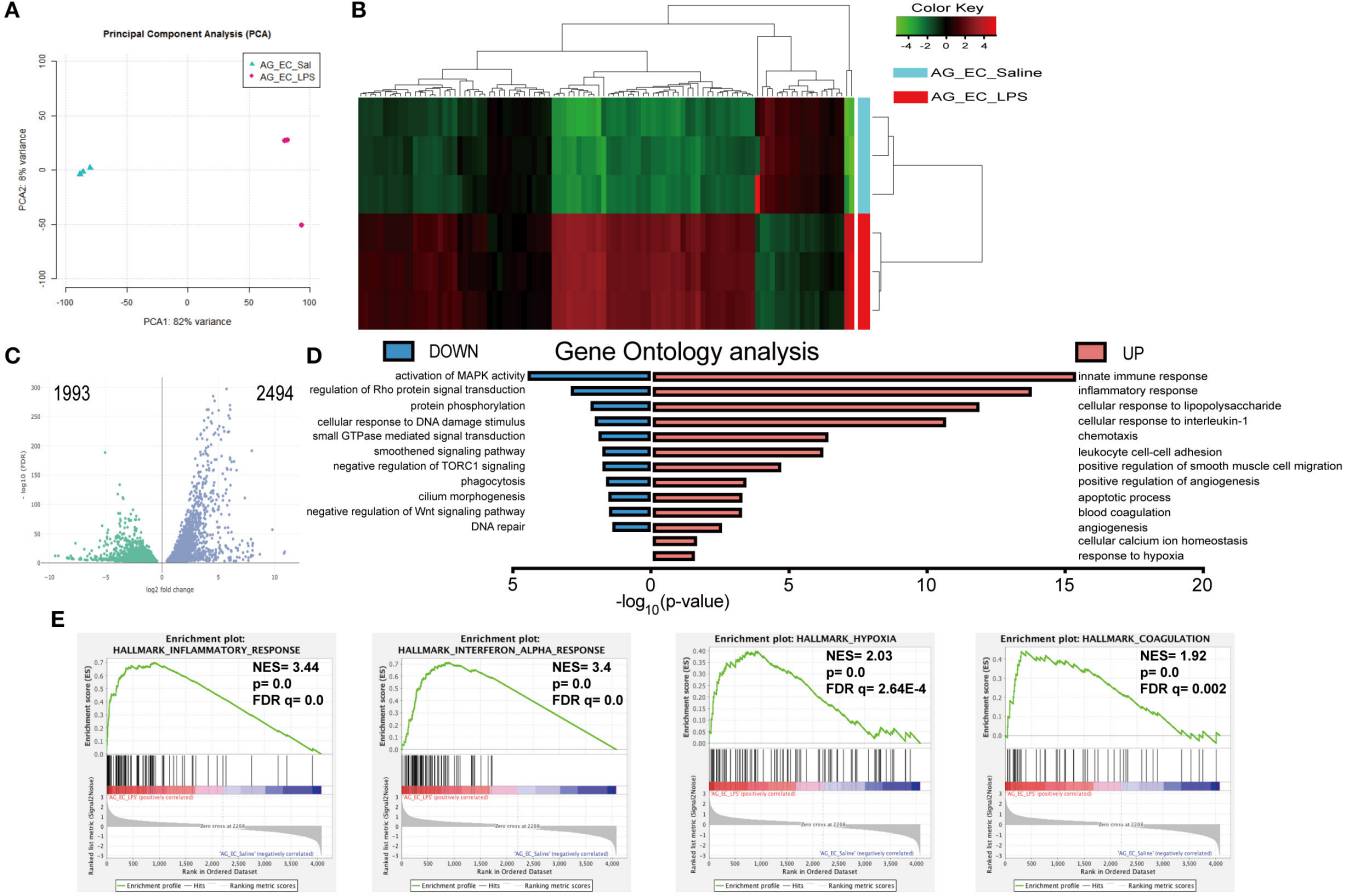
Bioinformatics analysis of the transcriptomes of the adrenal microvascular endothelial cells isolated from saline- and LPS-treated mice. Logarithm transformed counts from RNA-Seq dataset of adrenal microvascular endothelial cells isolated from saline- and LPS-treated adrenal glands were computed for sample correlation or variance by PCA analysis. Percentages in the PCA analysis axis indicated the proportional variance explained by each PC. LPS samples were labeled in red and Saline samples were labeled in blue that represented as condition located in the upper left part of the plot **(A)**. Unsupervised hierarchical clustering of the most 100 up- and down-regulated genes were computed by Euclidean distance clustering and expression heatmap with normalized raw z-scores. The labeling of Condition and DE were adapted as previous panels. **(B)** A volcano plot was depicted with –log10(FDR) against log2FoldChange of all 4487 significantly altered genes that were detected from the RNA-Seq dataset. Differential expression (DE) of genes was colored with green and blue, regarding 1,993 down-regulated and 2,494 up-regulated genes, respectively. Significance of differential gene expression was determined with adjusted *p*-value (p adj.) < 0.05 and counts > 50 **(C)**. Pyramid plot with bidirectional –log10 (*p*-value) demonstrated the involved gene ontology (GO) terms and pathways from submitted UP- or DOWN-regulated gene lists. The EASE score of *p*-value < 0.05 was set by DAVID software. Identical color code was used for showing UP and DOWN groups. *N* = 3 for each condition **(D)**. Representative 4 significantly enriched gene sets from GSEA analysis of 4,487 significantly changed genes in the RNA-Seq dataset. Enrichment plots comparing LPS toward Saline were depicted with the following sets of genes: **(A)** Hallmark Inflammatory response. **(B)** Hallmark Interferon-alpha response, Hallmark Hypoxia and Hallmark Coagulation **(E)**. Comparison of samples, NES, nominal *p*-value, and FDR q-value were determined by the GSEA software and were indicated within each enrichment plot.

Transcriptomic changes triggered by sepsis in the adrenal microvascular cells were evaluated by RNA sequencing using endothelial cells of mice that were injected either with physiological saline (control group) or bacterial LPS (SIRS group) for 3 h according to schema presented in Figure 1A. Principal component analysis (PCA) of our RNAseq results demonstrated a high separation of transcriptome patterns between both analyzed groups (PC1, 82 % of variance) and a very low variance among the biological replicates within each group (PC2, 4%), which suggest a clear separation of both analyzed experimental groups (Figure 2A).

We have next compared 11,137 differentially expressed genes identified from both groups with the DESeq2 package based on the following threshold—false detection rate (FDR < 0.01) and *p*-value < 0.05. As depicted in Figure 2C in the form of a volcano plot, systemic administration of LPS resulted in significant downregulation of 1,993 and upregulation of 2,494 genes in adrenal vascular cells. We have next performed unsupervised hierarchical clustering of the top 100 highest up- and down-regulated genes and found that LPS induced a profound and clear differential gene expression patterns in the adrenal endothelial cells with a tight clustering between both experimental groups (Figure 2B). Further analysis of the 100 upregulated genes demonstrated a high enrichment of genes involved in the regulation of inflammation (Table 1). In particular, LPS induced multiple chemokines such as members of the C-X-C motif ligand (Cxcl) family (Cxcl10, and Cxcl11), members of chemokine family characterized by (C-C motif) ligand including Ccl2, Ccl5, or Ccl7, and colony-stimulating factor 3 (G-CSF) and 2 (GM-CSF). Other top induced genes by LPS were those involved in increased leukocyte recruitment including P- and E-selectins and Vascular cell adhesion protein 1) and in inflammation such as IL-6, cyclooxygenase 2 (Ptgs2), sphingosine kinase 1 (Sphk1), and procoagulant gene encoding for plasminogen activator inhibitor type 1, member 1 (Serpine 1). In addition, endotoxemia resulted in the upregulation of multiple interferon-inducible genes such as an interferon-induced protein with tetratricopeptide Repeats (Ifit1-3), MX Dynamin Like GTPase 1 (Mx1) and Interferon regulatory element 5 (Irf5). Besides initiation of inflammatory genes endothelial cells initiate an anti-inflammatory program represented by Acod1 (Aconitate decarboxylase 1) gene, which is a negative regulator of TLR-signaling (28).

**Table 1:** Representative top 100 up- and downregulated genes from RNA-Seq analysis (related to Figure 2).

	**Gene ID**	**log_**2**_FC**
**UP-REGULATED**		
**Chemokines**	Csf3	10.89
	Ccl2	9.57
	Cxcl10	8.06
	Cxcl11	7.96
	Ccl11	7.67
	Csf2	7.41
	Ccl7	7.35
	Ccl5	7.18
**Leukocyte adhesion**	Selp	8.67
	Sele	6.13
	Vcam-1	6.40
	Icosl	5.8
**Angiopoiesis**	Pdgf	5.84
	Angpt2	5.72
**Inflammation**	IL6	9.79
	Sphk1	7.59
	IL27	7.18
	Ptgs2	7.1
	IL1rn	6.37
	Acod1	6.56
	Serpin1	6.44
	CD274	5.85
	Saa3	5.71
**INF-induced genes**	Mx1	8.06
	Ifit2	6.11
	Ifit3	5.99
	Ifit1	5.85
	Irf5	5.78
**DOWN-REGULATED**		
**Wnt & VEGFR signaling**	Ptk7	−8
**Apoptosis &Autophagy**	Dapk2	−4.96
**Development**	Shh	−5.93
**Cell junctions**	Rapsn	−8.44
	Dsc2	−6.1
	Zc4gz	−5.42
	Kcnb1	−4.63
	Pcdh12	−4.21

Among the most downregulated genes were those regulating protein phosphorylation, as well as genes involved in the development and vascular endothelial growth factor receptor (VEGFR) signaling, such as Sonic hedgehog (Shh) and Protein tyrosine kinase 7 (Ptk7), respectively (Table 1). The other downregulated genes found were those involved in apoptosis and autophagy including Death associated protein kinase 2 (Dapk2) and genes controlling cell junctions such as Receptor associated protein of the synapse (Rapsn) or Desmocollin 2 (Dsc2).

Functional analysis based on gene ontology identified the innate immunity, inflammation, cellular responses to cytokines and regulation of infiltration, as the most upregulated pathways induced by LPS in the microvascular adrenal endothelial cells (Figure 2D). Other highly enriched pathways were angiogenesis, apoptosis, hypoxia, and blood coagulation processes (Table S1). Subsequently, LPS suppressed genes regulating protein phosphorylation, activation of MAPK- and Rho-mediated signal transduction, smoothened signaling, cilium morphogenesis and DNA repair (Figure 2D and Table S2).

Finally, we have analyzed the potential contribution and interconnection of all significantly altered genes by a gene set enrichment analysis (GSEA) method. Based on the following criteria (nominal *p*-value < 5% and the false detection rate, FDR below 25%), we have found 22 gene sets that were positively enriched and none from 13 gene sets initially found to be downregulated by LPS. Four of the most induced gene sets by the LPS treatment are presented in Figure 2E. Those were gene sets involved in the inflammatory response (normalized enrichment score, NES = 3.44, *p*-value = 0.0 and FDR *q* = 0.0), response to interferon-alpha (NES = 3.4, *p* = 0.0 and FDR *q* = 0.0), the hypoxia (NES = 2.03, *p*-value = 0.0 and FDR *q* = 2.64 E-4) and coagulation pathway (NES = 1.92, *p*-value = 0.0 and FDR *q* = 0.002).

### Verification of RNA Sequencing Results

**Figure 3:**
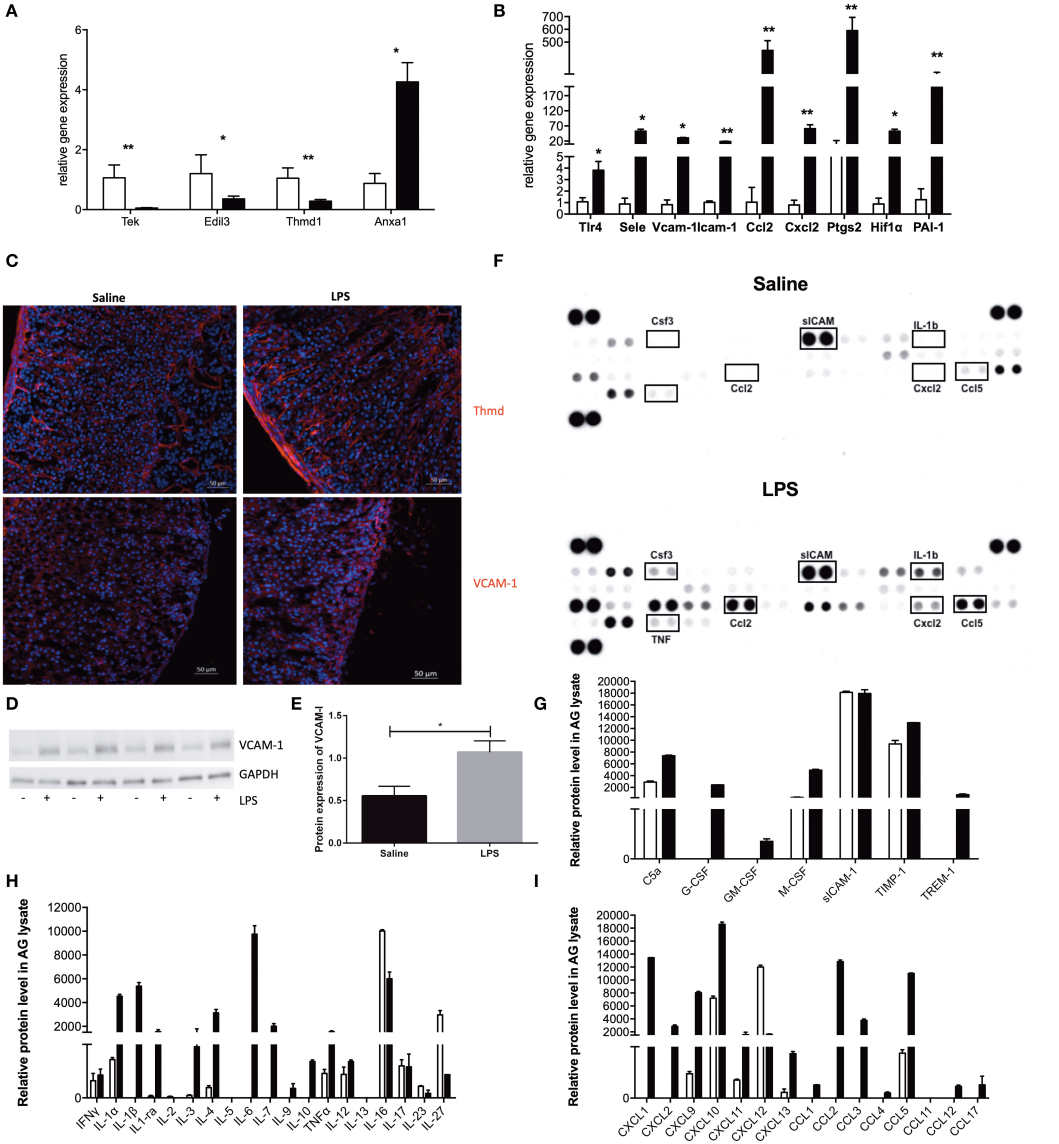
mRNA and protein verification of RNA sequencing analysis. Real-time PCR (qPCR) verification of expression of endothelial-specific genes **(A)** and expression of some of the top 100 most altered genes **(B)** with specific primers. Relative gene expression was determined by the ΔΔCt method with normalization of 18S ribosomal RNA. Saline-treated samples were depicted with open bars, and LPS-treated samples were depicted with black filled bars. *N* = 5 and statistics were calculated with the two-tailed non-parametric Mann-Whitney test. Values are mean with SEM. *p < 0.05, **p < 0.01. Images of fluorescent staining of thrombomodulin (Thmd) and Vascular adhesion molecule 1 (VCAM-1) **(C)** in adrenal tissue sections of mouse adrenal gland isolated from either Saline or LPS treated mice. Thmd and VCAM-1 were visualized using secondary antibodies coupled to Cy3 dye (red color). Western blot evaluation of VCAM-1 expression in adrenal glands isolated from mice treated either with Saline or LPS (*N* = 4) **(D)**. Graphic representation of the VCAM-1 and GAPDH protein expression quantified in ImageJ Software based on the densitometric evaluation of protein bands **(E)**. Results and evaluation of dot-blot based protein array **(F)** of different inflammatory-related molecules **(G)**, cytokines **(H)** and chemokines **(I)**. Saline-treated samples were depicted with open bars, and LPS-treated samples were depicted with gray filled bars. For each experimental group, two adrenals from three different animals (*N* = 6) were pooled and results are presented as double replicate.

In order to validate our RNA sequencing data, we have sorted the adrenal microvascular endothelial cells from mice that received either saline or LPS (5 mice per group) and verified the expression of some genes representing each identified pathway by real-time PCR (Figure 3).

We have first evaluated the expression of endothelial-specific genes that were previously reported to be regulated either positively or negatively by LPS action. In particular, we could confirm LPS-reduced mRNA expression of the Tek gene (encoding for an Ang receptor, Tie2), Edil3 gene (the EGF like repeats and discoidin domains 3, Del-1) and Thmd1 gene encoding for thrombomodulin. At the same time endotoxin highly upregulated Anxa 1 gene encoding for annexin A1 (Figure 3A). After reconfirming the identity of our sorted cells, we have analyzed the mRNA expression of genes identified by our gene ontology and the GSEA analysis. As presented in Figure 3B, we have found that LPS strongly induced expression of genes involved in the inflammatory response hallmark, such as toll-like receptor 4 (Tlr4), P- and E-selectins, Vcam-1 and an intracellular adhesion molecule−1 (Icam-1), chemokines (Ccl2, Cxcl2) and gens involved in prostaglandin synthesis e.g., cyclooxygenase 2 (Cox2, Ptgs2). Additionally, we have also validated the upregulation of genes encoding for hypoxia-inducible factor 1 alpha (Hif-1α), a gene representing the hypoxia hallmark, and the Serpine1 gene encoding for plasminogen activator inhibitor type 1, member 1 (PAI-1), which protein is known to inhibit fibrinolysis and therefore promote tissue coagulation.

In addition, to mRNA analysis, we have also studied the effect of LPS on the protein level using immunofluorescence, western blot and dot blot techniques in the whole adrenal gland. We have first looked at VCAM-1 expression in adrenal gland tissue after LPS administration because of its inducible character. As presented in Figure 3D, a strong induction of this adhesion molecule could be observed in the adrenal vascular cells after LPS administration (Figure 3C). Furthermore, we have additionally quantified (Figure 3D) this VCAM-1 induction by densitometrical analysis of western blot (Figure 3E). We have also studied the expression of thrombomodulin protein in adrenal tissue expression after LPS stimulation. On contrary to VCAM-1 however, LPS injection has not changed Thmd protein expression (Figure 3C). This result could imply that 3 h are too early to investigate a change in thrombomodulin protein levels, and later time points should be investigated.

As a part of the adrenal microenvironment, adrenal microvascular endothelial cells are in constant contact with intraadrenal produced pro-inflammatory mediators especially during systemic inflammation initiated by LPS. In addition, based on the above-presented transcriptome analysis, the adrenal endothelial cells may not just be a passive target of those inflammatory mediators, but also a substantial contributor to their production. Our analysis presented in Figure 3F revealed that during LPS-induced systemic inflammation, a significant induction of pro-inflammatory (IL-1α, IL-1β, TNF-α, IL-3, or IL-6) and anti-inflammatory (IL-4, IL-10, IL-1ra) cytokines were found. Whereas, the expression of others is either unchanged (IFN-γ, IL-17) or not detected at all (IL-5 and IL-13) Figure 3H. Among multiple chemokines detected the expression of CXCL1, CXCL2, CXCL9, CXCL10, CXCL12, CXCL13, CCL2, CCL3, CCL5 proteins were found to be strongly upregulated by LPS. This result corresponds to the induction of Cxcl2, Ccl2 verified by the Q-PCR or other chemokines, which expression was shown to be highly induced in adrenal microvascular endothelial cells by LPS according to our RNA-sequencing analysis (such as Cxcl10, Cxcl11, or Ccl5) presented in Figure 3I. Moreover, we have also observed a strong induction of G-CSF (CSF3) protein (Figure 3G), which was the highest upregulated gene found by RNA sequencing (Table 1). The latter observation is also in accordance with the literature, reporting that the microvascular endothelial cells are the main source of this chemokine.

## Discussion

It is generally accepted that endothelial cell dysfunction occurs progressively during sepsis and contributes to the pathophysiological function of many organs (14, 15). More importantly, a positive correlation between endothelial dysfunction and increased mortality rate of patients with sepsis was reported (29). Less is known, however, whether a similar situation occurs also in the adrenal glands.

In the present study, we aimed to investigate LPS-induced damage of the mouse adrenal microvascular cells *in vivo* using next-generation sequencing and protein analysis. To this end, we analyzed the transcriptomic changes in the endothelial microvascular cells isolated from mice 3 h after systemic administration of bacterial LPS. We have chosen LPS-model of systemic inflammation because of various reasons. In particular, LPS was shown to mimic an initial fulminant stage of Gram-negative sepsis induced by meningococcal infection in humans (30). Furthermore, it has the ability to induce a robust transcriptomic response in multiple organs in a highly reproducible manner. Finally, LPS-induced changes in the adrenal gland transcriptome were also previously verified by us in a more clinically relevant model of sepsis—a cecal-ligation and puncture (CLP)-induced peritonitis (12). We have chosen a 3 h time point for analysis because of the transient nature of an *in vivo* action of LPS, especially regarding the transcriptomic response.

Our results from RNA sequencing supported by bioinformatics analysis demonstrated that LPS induced multiple genes and enriched gene sets that are involved in the control of the innate immunity and tissue inflammation. Those include pathogen recognition receptors, cytokines, chemokines, immune cell modulators, and adhesion molecules.

We have found an increased expression of some key pattern recognition receptors, including Tlr2, Tlr3, and Tlr4 along with nuclear oligomerization domain member 2 (Nod2) in the adrenal microvascular endothelial cells. Expression of those receptors sensitizes endothelial cells to the action of several Gram-negative (Tlr4) and positive (Tlr2) bacteria along with several viral infections (Tlr3). The expression of the LPS receptor (Tlr4) was additionally verified by real-time PCR in a separate group of mice. This result is also in accordance with available literature demonstrating the expression of several TLRs in microvascular endothelial cells (31).

In our previous studies, we have shown that activation of TLR4 triggers the expression of multiple cytokines and chemokines in the adrenal gland on the mRNA (12) and protein levels (32). Furthermore, we have found that inactivation of immune but not adrenocortical TLR-signaling could partially decrease this effect (32). Based on this observation, we have postulated that other cells of the adrenal microenvironment, potentially the endothelial cells, may be involved (4). In order to validate this hypothesis, in the present study we have compared the LPS induced cytokines in the adrenal gland with those found only in the adrenal endothelial cells. We have found that systemic administration of LPS increased expression of TNF-α, IFN-α, IFN-γ, IL-12, IL-1α, IL-1β, IL-7, IL-11, IL15, and IL-33 cytokines in the adrenal glands, whereas, at the same time upregulation of only IL-6, IL-27, and IL-15 cytokines was found in endothelial cells. This result suggests that adrenal endothelial cells may contribute to the local inflammation by upregulating IL-6. Limited information is available regarding the transcriptomic analysis of the other microvascular cells during sepsis. However, the existing *in vitro* experiments support our observation, showing that LPS induces mostly IL-6, diverse chemokines and growth factors from cultured endothelial cells (31, 33).

Although endothelial cells might not be substantially the main source of the cytokines during the sepsis, more importantly, those cells are the main target of pro-inflammatory mediators. Indeed, vascular cells are known to express receptors for various pro-inflammatory mediators that are being released into the circulation during sepsis, such as cytokines, chemokines, growth factors, reactive oxygen species (3). Therefore, we have next studied the pro-inflammatory milieu induced by LPS at a 3 h time point in the adrenal gland using a multi cytokine protein array. This assay confirmed a strong induction of IL-6, but also TNF-α, IFN-γ, IL-13, IL-7, and IL-1α or IL-1β cytokines in the adrenal gland lysates.

All those cytokines were shown to contribute to sepsis-mediated activation of the endothelium leading to its increased permeability, altered vascular tone, promotion of a procoagulant state and leukocyte adhesion (13). In order to study whether adrenal microvascular cells are also activated during endotoxemia, we have compared the expression of diverse chemokines and adhesion molecules between the adrenal gland (12) and isolated endothelial cells. The results of this comparison suggest that the adrenal endothelium may be the main contributor of Csf3, Ccl2, Csf2, and Ccl5, whereas the expression of Cxcl11, Ccl4, or Cxcl5 chemokine can originate also from other cells of the adrenal microenvironment. In addition, the results of our protein array analysis confirmed that indeed the adrenal microvascular endothelial cells contribute substantially to the secretion of CSF (G-CSF), CCL2 and CCL5 chemokines. This observation is in accordance with recent studies using an endothelial cell-specific inactivation of TLR4 signaling. In those mice, a significant decrease in infection-induced plasma CSF3 levels and related lack of emergency granulopoiesis from the bone marrow was observed (34). Furthermore, a significant reduction of LPS-induced expression of adhesion molecules, leukocyte infiltration, and vascular permeability was observed (35). The latter observation was additionally supported in a TNF-induced endothelial damage model, in which inactivation of endothelial TLR signaling decreased plasma CCL5, IL-6, and iNOS levels (36). These observations collectively suggest that LPS activates adrenal endothelium, and promotes expression and secretion of several chemokines (CCL2, CCL5, and CSF3) and adhesion molecules from those cells.

Besides attracting leukocytes, the locally expressed chemokines are also involved in the activation of those immune cells on the endothelium surface (37). Infiltration of immune cells into organs requires multiple steps, including selecting-mediated rolling, chemokine-induced activation, adhesion molecule-mediated firm adhesion, and transmigration through endothelium govern by junctional adhesion molecules and CD31 (37). In the absence of pathogens, the surface of the endothelial cells is rather antiadhesive. Particularly, in the adrenal gland, this process is governed by the secretion of Del-1 protein, which is an antagonist of leukocyte adhesion by preventing the ICAM-1-LFA1 interaction (38). Our study revealed that LPS rapidly induces expression of genes encoding for the P- and E-selectins as well as ICAM-1 and VCAM-1 adhesion molecules. We have verified those changes by real-time PCR and in the case of VCAM-1 also on the protein level in the adrenal gland. Together with decreased expression of Tek and Edil3 genes, which suggest increased vascular permeabilization, increased expression of adhesion molecules may promote the infiltration of immune cells into the adrenal gland. Indeed, in our previous studies, we demonstrated that intraperitoneal injection of LPS to mice results in a rapid infiltration of immune cells into the adrenal gland and that Del-1 deficiency aggravated this process (18). Leading to enhanced apoptosis of adrenal cells, and reduced corticosterone production (18).

An increasing number of studies demonstrate the sepsis-mediated increase in vascular leakiness and their persistent pro-coagulative phenotype, which leads to a progressive hypooxygenation of the affected tissues and can contribute to multi-organ failure (14). In particular, in a study utilizing the cecal ligation and puncture (CLP) model of experimental sepsis, temporary inhibition of the blood flow was demonstrated in skeletal muscle, which led to an improper oxygen distribution and local hypoxia (39). Whether hypoxia can also contribute to the adrenal gland dysfunction is unknown. In our study, we have observed a strong upregulation of hypoxia-inducible factor 1-alpha (Hif-1α), angiopoietin-like 1 (Angptl1) and glycolysis hexokinase 1 and 2 (HK1, 2) in microvascular endothelial cells, which are major hypoxia-related genes. Out of those, we have additionally verified the upregulation of Hif-1α by real-time PCR. On contrary to the results from a transcriptomic analysis of an intact adrenal gland (12), we have not observed any differences in the expression of von Hippel-Lindau tumor suppressor (Vhl) gene in the isolated adrenal endothelial cells. The potential reason of this observation may relate rather to the pro-inflammatory effect of the HIF-1α stabilization and activation induced by LPS and not hypoxia itself. Although activation of HIF-1α was also reported under normoxic conditions preceding the hypoxic conditions (40).

A hallmark of sepsis-induced vascular dysfunction is an increased permeability (13). The endothelial leakage can be initiated either by a loss of protective glycocalyx layer (41), or disturbance in Angiopoietin2 (Angpt2)-Tie2 interaction. In our study, we have investigated the second mechanism. In particular, we have found that LPS injection upregulates the Angpt2 gene in adrenal microvascular cells. This may analogously to the other organs, lead to the increased occupation of Tie2 receptors (42) and increased VEGF-mediated vascular permeability (43). At the same time, LPS-reduced expression of Tek gene, thereby additionally contributing to the Tie2-Anpt2 dysbalance. The loss of this critical tyrosine kinase receptor expressed by endothelial cells was reported recently in diverse critical illnesses including human sepsis, as was correlated with endothelial dysfunction (44).

A well-established hallmark of sepsis is dysregulation of a balance between coagulation and fibrinolysis, which process may lead to disseminated intravascular coagulation (DIC), and subsequently to multiple organ failure. The adrenal gland is an extremely vascularized organ that receives a 10-times higher amount of blood supply in regards to its weight (45, 46), which sensitizes it to hemorrhages. In fact, singular or bilateral adrenal bleedings are being regularly reported, especially after trauma or in patients with meningococcal sepsis (47, 48).

In the present study, systemic inflammation was associated with a strong upregulation of the tissue factor gene, which is one of the main inducers of coagulation (16). Furthermore, we have also observed a strong upregulation of PAI-I (encoded by the Serpin1 gene), which inhibits plasminogen formation and fibrinolysis. Those observations particularly in a view of decreased thrombomodulin gene (thbd1) expression and reported fibrin deposition (49), suggest that LPS increases potentially also the risk of adrenal vascular coagulation. However, a longer time point should be considered when analyzing the adrenal coagulation, as 3 h were not sufficient to alter the protein expression of this important cofactor of protein C activation in the adrenal tissue.

In order to counterbalance the mostly pro-inflammatory effect of LPS on the adrenal microvascular cells, several protective and inflammation resolving factors are subsequently upregulated. Those include first of all IL-4, and IL-10 cytokines, as well as genes encoding for cis-aconitate decarboxylase (Acod1), annexin 1 (Anxa1) and sphingosine kinase 1 (Sphk1). Upregulation of the Acod1 was reported to antagonize Toll-like receptors (TLRs)-mediated inflammatory innate response (28), whereas induction of annexin 1 was found to mediate the anti-inflammatory action of glucocorticoids (50). Furthermore, as a part of the homeostatic action, adrenal endothelial cells highly upregulated sphingosine kinase 1 (Sphk1) that phosphorylates sphingosine to sphingosine-1-phosphate (S1P), which factors are known to stabilize the endothelial barriers (51). Whether endothelial dysfunction leads to adrenal gland dysfunction, will depend on the delicate balance between pro and anti-inflammatory pathways.

Altogether, this is the first study presenting the LPS-induced global changes in the transcriptome by the adrenal microvascular cells. The results of RNA sequencing and subsequent bioinformatics analysis and protein verification revealed that adrenal vascular endothelial cells are not only involved in the maintenance of the adrenal gland microenvironment but are also active players in pathogen-induced adrenal pathophysiology. Moreover, our results suggest that the damage to the microvascular cells may be an important step preceding the adrenal gland dysfunction, however additional experiments that are ongoing are needed to verify this statement.

## Data Availability Statement

Sequencing data are available at the Gene Expression Omnibus database (http://www.ncbi.nlm.nih.gov/geo/) under the following corresponding GSE accession number: GSE139134. Statistical analysis of relative gene expression results and densitometry data were conducted using a non-parametric Mann-Whitney U test in GraphPad Prism Version 6 software (GraphPad Software Inc., USA).

## Ethics Statement

The animal study was reviewed and approved by LANDESDIREKTION SACHSEN.

## Author Contributions

L-SC, WK, and SS conducted research. L-SC, WK, SS, and GM analyzed data. WK, L-SC, and SB wrote the manuscript.

### Conflict of Interest

The authors declare that the research was conducted in the absence of any commercial or financial relationships that could be construed as a potential conflict of interest.
