# Supplementary material for: Transcriptional Analysis of Sepsis-Induced Activation and Damage of the Adrenal Endothelial Microvascular Cells
Source: Front Endocrinol (Lausanne). 2020 Jan 22;10:944. doi: 10.3389/fendo.2019.00944 (PMC6987315; doi:10.3389/fendo.2019.00944)
Supplement: Supplementary file 1 [file Data_Sheet_1.PDF]

Table S1. Up-regulated gene lists involved in functional annotation analysis

GO or KEGG ID	Description	p-value	Gene ID
**GO:0045087**	innate immune response	5.52E-16	Oas3/Zbp1/Fcgr1/Irgm1/Ddx58/Acod1/Oas1a/Ifit2/Anxa1/Pml/Sp110/Nlrc5/Csf1/Ripk2/Mx1/Oasl2/Samhd1/Oasl1/Ifit3/Isg20/Ifit1/Il23r/Tlr4/Mx2/Herc6/Oas1b/Jak2/Rsad2/Eif2ak2/Irf5/Tlr2/Iigp1/Adar/Ifih1/Il27/Casp4/Cfb/Tlr3/Ifitm3/Rnf135/Irf7/Dhx58
**GO:0006954**	inflammatory response	1.88E-14	Cxcl1/Sema7a/Ccl11/Zc3h12a/Tnfrsf9/Ccl4/Tnfaip3/Thbs1/Acod1/Anxa1/Ccl7/Csf1/Ccl5/Ccl3/Gbp5/Ptgs2/Cxcl5/Il23r/Tlr4/Cxcl11/Jak2/Ccl9/Tnfrsf11b/Sphk1/Olr1/Tnip1/Tlr2/Cd40/Cxcl10/Il27/Casp4/Cxcl2/Tlr3/Il6/Selp/Sele/Ccl2
**GO:0071222**	cellular response to lipopolysaccharide	1.74E-12	Zc3h12a/Tnfaip3/Acod1/Stat1/Serpine1/Gbp10/Tnip3/Gbp2/Litaf; Gm9861/Ripk2/Sbno2/Csf3/Cmpk2/Csf2/Tlr4/Gbp6/Jak2/Cxcl16/Pde4b/Icam1/Fcgr4/Cd40/Cxcl10/Cxcl2/Il6/Cd86/Ccl2
**GO:0071347**	cellular response to interleukin-1	2.52E-11	Ccl11/Zc3h12a/Ccl4/Ccl9/Hif1a/Acod1/Icam1/Serpine1/Sox9/Saa3/Cxcl2/Ccl7/Il6/Ccl5/Ccl3/Myc/Ccl2
**GO:0006935**	chemotaxis	4.53E-07	Ccl11/Ccn1/Ccl4/Cxcl11/Ccl9/Cxcl16/Lgals9/S1pr1/Cxcl10/Cxcl2/Ccl7/Ccl5/Ccl3/Ccl2/Cxcl5
**GO:0007159**	leukocyte cell-cell adhesion	7.14E-07	Ccl5/Vcam1/Olr1/Tnip1/Icam1/Selp/Sele/Itga5
**GO:0014911**	positive regulation of smooth muscle cell	2.37E-05	P2ry6/Itgb3/Ccl5/Itga2/F3/P2ry2/Myc/Pdgfb
**GO:0045766**	positive regulation of angiogenesis	4.27E-04	Ccl11/Itgb3/Pgf/Zc3h12a/Ccl5/Angpt2/Hif1a/Sphk1/Thbs1/F3/Serpine1
**GO:0006915**	apoptotic process	5.85E-04	Zc3h12a/Tnfrsf9/Tnfaip3/Xaf1/Ifit2/Stat1/Pml/Sp110/Litaf; Gm9861/Cflar/Ripk2/Pim1/Daxx/Plekhf1/Clic4/Bid/Acvr1c/Rmdn3/Fap/Tnfrsf11b/Map3k8/Nek6/Ppp1r13l/Dnase1l3/Tnfrsf12a/Ppp2r2b/Birc3
**GO:0007596**	blood coagulation	6.23E-04	Procr/Wee1/Tfpi2/Enpp4/F3/Pdgfa/Pdgfb/Mafk/Maff
**GO:0001525**	angiogenesis	0.00320754	Rnf213/Clic4/Zc3h12a/Angpt2/Myh9/Fap/Hif1a/Serpine1/S1pr1/Pgf/Tnfrsf12a/Pdgfa/Ccl2/Ptgs2
**GO:0006874**	cellular calcium ion homeostasis	0.02600018	Itgb3/Rmdn3/Ccl5/Ccl3/Vdr/Ccl2/Cd40
**GO:0001666**	response to hypoxia	0.03086059	Pgf/Sod2/Angpt2/Vcam1/Hif1a/Itga2/Plat/Tlr2/Ccl2

Table S2. Down-regulated gene lists involved in functional annotation analysis

GO or KEGG ID	Description	p-value	Gene ID
**GO:0000187**	activation of MAPK activity	3.59E-05	Tgfb3/Ntf3/C1qtnf2/P2rx7/Plce1/Map2k6/Efna1/Igf1/Gab1/Cspg4
**GO:0035023**	regulation of Rho protein signal transduction	0.00133328	Plekhg5/Als2cl/Arhgef10/Rasgrf2/Vav3/Arhgef28/Prex2/Prex1
**GO:0006468**	protein phosphorylation	0.00693181	Gas6/Pick1/Camkv/P2rx7/Cdk20/Camkk1/Pdk2/Pkn3/Dapk2/Camk1/Map4k2/Aatk/Mast3/Ptk7/Rps6ka1/Cdc25b/Map2k6/Stk10/Rps6ka5/Mapk12/Chek2/Hunk/Tek
**GO:0006974**	cellular response to DNA damage stimulus	0.00978464	Pole/Nthl1/Apbb1/Polm/Casp9/Mutyh/Rpa3/Fbxo31/Slx1b/Fancb/Prkdc/Fancg/Sirt4/Stxbp4/Vav3/Chek2/Rad51c/Msh2
**GO:0007264**	small GTPase mediated signal transduction	0.01348958	Rasgrp2/Rab27a/Arl4d/Plce1/Rhebl1/Rasgrf2/Vav3/Dock6/Rgl3/Arhgap18/Rab7b/Rab3a
**GO:0007224**	smoothened signaling pathway	0.01835692	Cc2d2a/Shh/Evc/Smo/Tmem231/Tbc1d32
**GO:1904262**	negative regulation of TORC1 signaling	0.01853114	Sesn1/Castor2/Castor1
**GO:0006909**	phagocytosis	0.02539007	Gas6/Gulp1/Pip5k1c/Myo7a/Pld4
**GO:0060271**	cilium morphogenesis	0.03098422	Ift81/Cdc14a/Cc2d2a/Ttll1/Fbf1/Cby1/Ccdc28b/Tmem231/Tbc1d32
**GO:0030178**	negative regulation of Wnt signaling pathway	0.03224085	Dkk2/Shh/Cxxc4/Cby1/Rnf43
**GO:0006281**	DNA repair	0.04308728	Pole/Prkdc/Fancg/Nthl1/Polm/Eepd1/Mutyh/Rpa3/Chek2/Rad51c/Msh2/Slx1b/Fancb
